# Acute Extensive Deep Vein Thrombosis After Heterogeneous Administration of Moderna mRNA Booster Vaccine: A Case Report

**DOI:** 10.7759/cureus.25779

**Published:** 2022-06-09

**Authors:** Farrah Alarmanazi, Bushra A Bangash, Lokesh Lahoti, Banu Farabi

**Affiliations:** 1 Internal Medicine, Saint Peter’s University Hospital, New Brunswick, USA

**Keywords:** acute deep vein thrombosis, moderna mrna vaccine adverse effects, heterogenous vaccinations, booster vaccine, covid 19, sars-cov-2

## Abstract

Severe acute respiratory syndrome coronavirus 2 (SARS-CoV-2) struck the world in 2019 and led to the development of the multisystem coronavirus disease-2019 (COVID-19) causing a worldwide pandemic. Vaccines with boosters were developed due to novel mutations of SARS-CoV-2. Heterogeneous vaccination emerged with the perception that mixing vaccines can provide better protection. We present the case of a 68-year-old male patient who developed extensive acute deep vein thrombosis (DVT) of the left lower extremity, two weeks following the Moderna mRNA booster vaccine (mRNA-1273). His first two doses were AstraZeneca ChAdOx1-S [recombinant]. He was started on a heparin drip and prescribed rivaroxaban. We discuss the possible etiology of this DVT, the mechanism of action of the Moderna mRNA vaccine, the association of DVT with vaccine-induced inflammation, implications of heterogeneous vaccine combinations, and recommendations to advise people on possible thrombogenic adverse effects prior to mRNA vaccine administration.

## Introduction

The multisystem coronavirus disease-2019 (COVID-19) caused by severe acute respiratory syndrome coronavirus 2 (SARS-CoV-2) led to more than 385 million cases and six million deaths worldwide as of February 2022. Many companies started developing vaccines using highly advanced technologies. Different viral components were used, with more than 30 vaccines approved for emergency use worldwide. Only three vaccines are approved by the Food and Drug Administration (FDA) in the USA, two of these are mRNA-based vaccines which are Pfizer/BioNTech (BNT162b2) and Moderna (mRNA-1273). The mRNA vaccine technology is very recent with many studies supporting their high efficacy in generating an immune response against SARS-CoV-2, however, there is limited data on the adverse effects of these vaccines and long-term complications [1]. Moreover, with new viral variants emerging, booster vaccines were developed and heterogenous administration was encouraged as studies show an increased immunologic and inflammatory response using this approach. However, there is no available data on the possible complications of mixing vaccines [2].

Adverse effects are being continuously updated on the self-reported Vaccine Adverse Event Reporting System (VAERS) by the Centers for Disease Control and Prevention (CDC) and FDA. As of March 2022, the most common reported adverse effects of the Moderna mRNA vaccine are headache (16.62%), pyrexia (15.62%), fatigue (14.26%), and chills (12.44%). Although less commonly reported, deep vein thrombosis (DVT) made up 0.38% with 1,622 reported cases [3]. In this article, we discuss a case of extensive left lower extremity DVT in a 68-year-old male with no significant prior medical history, two weeks after receiving the Moderna mRNA booster vaccine. Our discussion details the mechanism of action of mRNA vaccines and how it induces a vigorous immunologic, inflammatory, and thrombogenic response that can lead to endothelial damage. This, in addition to our patient receiving the AstraZeneca vaccine six months prior, suggests that his immune and inflammatory response to the mRNA vaccine could have been exacerbated by mixing vaccines leading to an increased thrombogenic state and development of extensive DVT.

## Case presentation

**Figure 1:**
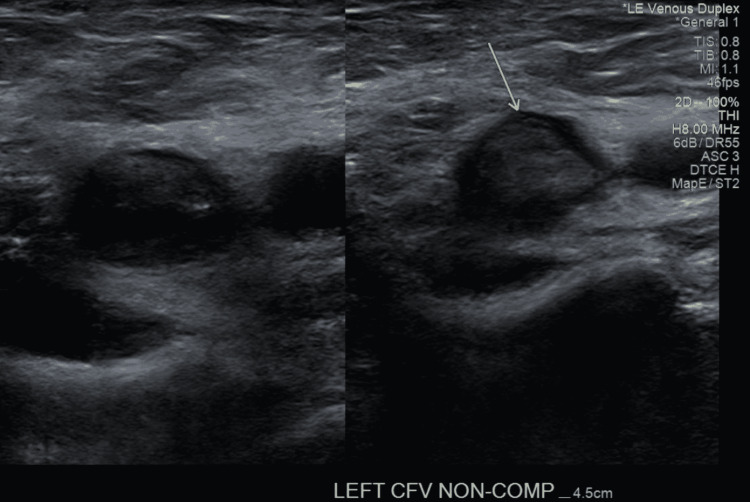
Venous duplex ultrasound of the left lower extremity showing a thrombosed non-compressible left common femoral vein.

**Figure 2:**
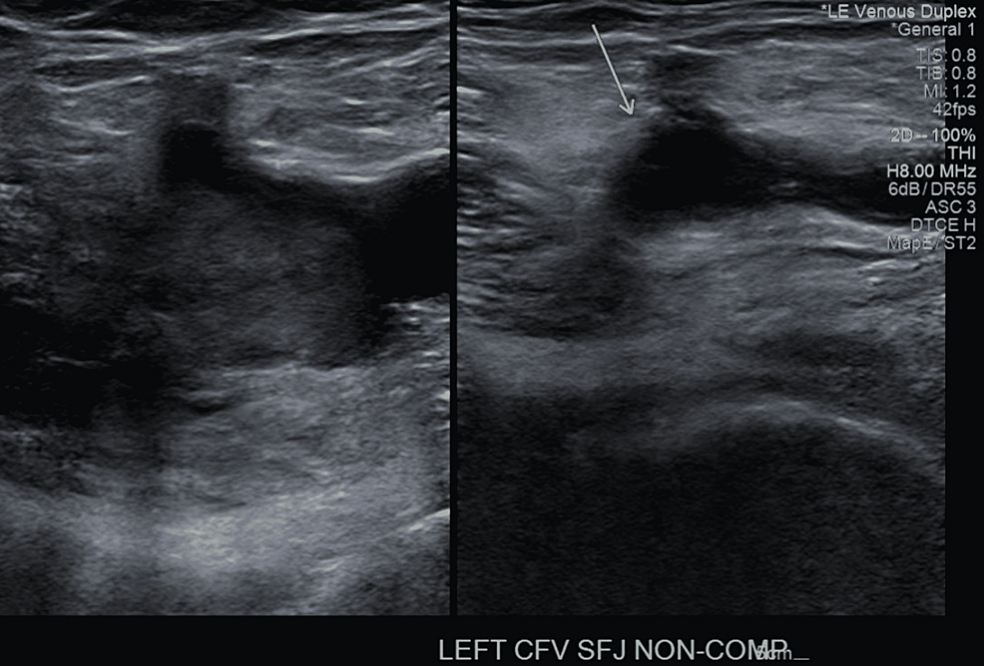
Venous duplex ultrasound of the left lower extremity showing a thrombosed non-compressible left saphenofemoral junction.

A 68-year-old man from Colombia was in his regular state of health before boarding a six-hour direct flight to New York. Within the second hour of the flight, he started feeling a constant sharp pain in his left lower extremity, associated with swelling and red discoloration. He was brought to the emergency department (ED) the same day by his family due to severe pain. The patient denied having any symptoms before boarding the plane. The patient took the Moderna mRNA COVID-19 booster vaccine two weeks prior to this episode. He finished his first two doses of the AstraZeneca COVID-19 vaccine more than six months prior to the booster without complications. The patient did not report recent trauma to the area and his past medical history was non-contributory, including no prior hospitalization, recent surgeries, DVT, pulmonary embolism (PE), COVID-19 infection, or malignancy. The patient had a colonoscopy seven years ago which was negative. No family history of DVT, malignancy or clotting disorders was present. The patient never smoked and did not use alcohol or illicit drugs. His medical conditions were pre-diabetes controlled with Metformin 1000 mg orally daily with HbA1c being <6.5%, hyperlipidemia controlled with Atorvastatin 40 mg orally daily, and hypertension controlled with Amlodipine-Valsartan 5 mg-160 mg orally twice daily, and Metoprolol tartrate 2.5 mg orally twice daily. On review of systems, he denied fatigue, fever, chills, nausea, vomiting, dizziness, headaches, shortness of breath, chest pain, palpitations, changes in bowel or bladder habits, numbness or tingling in the extremities, or joint pain. His vital signs were within normal limits upon admission including a pulse of 61 beats per minute, respiratory rate of 12 breaths per minute, a temperature of 98.5 degrees Fahrenheit, oxygen saturation of 98% at room air, and a body mass index (BMI) of 24.4 kg/m2. Pertinent findings included positive tenderness, erythema, and generalized pitting edema extending from the proximal thigh to the toes in the left lower extremity. While the right lower extremity was negative for edema, or skin changes, bilateral lower extremity pulses were diminished with a value of +2 in the left and right femoral, popliteal, dorsalis pedis, and posterior tibial veins. Grossly intact motor and sensory functions were present in the bilateral lower extremities. The patient was negative for an active COVID infection confirmed by a polymerase chain reaction test in the ED. Laboratory findings revealed a hemoglobin of 15.7 g/dL, hematocrit of 46.2%, platelet count of 173,000/mm3, an international normalized ratio (INR) of 0.96 s, a partial thromboplastin time (PTT) of 29 s, and a prothrombin time (PT) of 10.8 s. Thrombophilia workup was negative including prothrombin, the activity of Anti-Thrombin III, Factor V Leiden, Protein C, and S. Venous duplex ultrasound of the lower extremities showed evidence of acute, occlusive DVT involving the left iliac, common femoral, femoral, deep femoral, popliteal, gastrocnemius, posterior tibial, and peroneal veins (Figure 1). The superficial vein was also thrombosed involving the left great saphenous vein at the saphenofemoral junction (Figure 2). The right leg did not show any evidence of thrombus. He was started on an IV heparin drip immediately, using the activated partial thromboplastin time (aPTT) to monitor for the anticoagulation response. His pain improved the next day with stronger lower extremities pulses, measuring +2 bilaterally. Subsequently, he was discharged on 15 mg Rivaroxaban twice daily for 21 days and 20 mg once daily for three months with instructions to follow up in the out-patient clinic. Repeat venous duplex a month later showed no evidence of deep or superficial vein thrombosis of either lower extremity. Indicating that the extensive thrombus previously visualized in the left lower extremity appears to be resolved.

## Discussion

The scientific-technological advances in the 21st century allowed rapid and extensive studies to be conducted on the SARS-CoV-2 virus, and vaccines were developed. Targets of these vaccines included nucleic acids such as genetically modified DNA and mRNA, protein subunits, peptides, and recombinant proteins, non-replicating viral vectors, replicating viral vectors, virus-like particles, inactivated viruses, and attenuated viruses [1].

The Moderna vaccine is one of the three vaccines administered in the United States. Its mechanism of action is based on using mRNA isolated from the SARS-CoV-2 virus spike proteins. Prior case reports associated with Moderna vaccine administration included patients with DVTs to the upper extremities at the injection site or lower extremities with most being females who are predisposed to DVT due to higher estrogen states. Our case is unique in that our patient is a male, with no predisposing medical history. His pain and swelling developed within the second hour of his flight which represents the tendency for developing diffuse DVT even with short-term immobilization with the effect of Moderna vaccine precipitating intravascular thrombosis. Moreover, what differentiates this case from other reported cases of DVT post-vaccination with a first or second dose of the Moderna vaccine is that our patient’s DVT developed two weeks after the Moderna Booster vaccine. The booster was a heterogenous addition secondary to the first two doses being AstraZeneca.

The AstraZeneca vaccines have been associated with vaccine-induced immune thrombotic thrombocytopenia (VITT), which is a result of platelet factor-4 antibodies induced by adenoviral vector COVID-19 vaccines. Current data report that incidences of VITT are approximately 1/100,000 exposures [4]. However, our patient's clinical picture does not fit VITT since VITT presents with thrombocytopenia, and the platelet activation occurs in unusual venous and arterial sites such as cerebral venous sinus thrombosis. Our patient platelet count was within normal range and his DVT was in the left lower extremity which is a common site of thrombosis [5].

The Moderna vaccine mechanism of action starts with the mRNA segment that is attached to a nanoparticle which is injected intramuscularly into humans. Once in the body, the mRNA inserts into cells' cytoplasm, gets translated by the ribosomes and produces viral spike proteins. These proteins activate the immune system via expression on MHC I and MHC II complexes which activate immune response via T-cells, B-cells, macrophages, and dendritic cells. As a result of this activation, specifically, the T-helper cells, cytokines, and interleukins are produced which stimulate the B-cells to differentiate into plasma cells. Plasma cells activation leads to the production of a massive amount of antibodies against the viral spike proteins which leads to their destruction. At the same time, the interleukins induce T-cells to differentiate into memory cells that will be activated if future infection occurs [6].

This response mechanism is what makes mRNA vaccines effective, however, this process is based on the mRNA being translated by ribosomes. This translation occurs secondary to its entrance into the cell’s cytosol by binding to receptors called pattern recognition receptors (PRRs). Binding PRRs leads to the activation of a significant endothelial inflammatory response. This inflammatory response is due to the activation of Toll-like raptors (TLR) such as TLR3, TLR7, and TLR8, in addition to melanoma differentiation-associated protein 5 (MDA5) and retinoic acid-inducible gene-I (RIG-I). The massive inflammatory effect leads to multiple pro-inflammatory cascades being activated such as I-interferon (IFN) response and the nuclear translocation of the transcription factor nuclear factor (NF)-kB [7].

The up-regulation of pro-inflammatory cascades, and activation of endothelial cells, platelets, and leukocytes have the ability to trigger the coagulation cascade by inducing tissue factors (TFs). Such activation can trigger a pro-thrombotic state that can lead to DVT formation. There is growing evidence that suggests an association between inflammation and venous thromboembolism [8]. This raises concerns about DVT being a possible adverse effect of mRNA vaccines.

Investigations into the adverse effects of heterogeneous vaccine administration lack data and are still unknown. Some studies on the administration of mRNA vaccines as a second dose after the first dose of AstraZeneca ChAdOx1-S [recombinant] showed an increased level of neutralizing antibody levels and higher T-cell-mediated immune response compared to two doses of an mRNA vaccine. Even though this can be promising, it can also be concerning for increased inflammatory response and an increased risk of adverse effects (AEs). Since our patient’s mRNA vaccine was after two doses of the AstraZeneca vaccine, we suggest that the inflammation process induced by mRNA vaccines alone could have been exacerbated by the heterogeneous combination leading to the extensive DVT development in our patient’s left lower extremity [2].

We recognize that there is limited data on heterogenous vaccines AE and that this approach can be beneficial, especially with an increased number of people requesting vaccinations and a limited supply. However, since there are 1,592 cases of DVT reported via VAERS after the administration of the Moderna mRNA vaccine as of March 2022, we strongly recommend that patients receiving the Moderna mRNA vaccine should be advised about the increased risk of venous thromboembolism prior to taking mRNA vaccines [3].

We acknowledge the need for further investigation of unprovoked DVT causes in our case. These investigations include tests for antiphospholipid antibody syndrome and repeating the thrombophilia panel in four to six months since acute illness can cause misleading results. Our patient returned to his home country and was instructed to follow up there. 

## Conclusions

The mRNA vaccines have recently emerged as a highly efficacious technology, however, more data need to be collected on their adverse effects. The vigorous immunologic, inflammatory, and prothrombotic response to mRNA vaccine can possibly lead to extensive DVT even in patients with no predisposing factors. We argue that this response can be further exacerbated by mixing vaccines as presented in our case. We acknowledge the benefit of these vaccines as it outweighs the risks of being infected with COVID-19, and these reported AE should not discourage patients from getting vaccinated. We acknowledge the limitation of our conclusion as this is a single case report and the need for further studies. We recommend further data collection with regard to the adverse effects of heterogeneous vaccine administration and generating an advisory on the thrombogenic adverse effects if the data support a correlation with heterogenous vaccinations. We recommend further data collection with regard to the adverse effects of heterogeneous vaccine administration and generating an advisory on the thrombogenic adverse effects if the data support a correlation with heterogenous vaccinations. 
